# Spatially fractionated radiotherapy with PD-L1 blockade achieves superior antitumor efficacy and safety in a preclinical model of peritoneal metastasis

**DOI:** 10.1007/s00262-026-04505-w

**Published:** 2026-08-06

**Authors:** Sung Eun Lee, Changhoon Choi, Yeeun Kim, Taehyung Kim, Sung Jin Kim, Sang Hoon Jung, Jeong Il Yu

**Affiliations:** 1 Department of Radiation Oncology, Samsung Medical Center, Seoul, 06351 Republic of Korea; 2 Department of Radiation Oncology, Sungkyunkwan University School of Medicine, Seoul, 06351 Republic of Korea

**Keywords:** Gastrointestinal tumor, Peritoneal carcinomatosis, Spatially fractionated radiotherapy, Immune checkpoint inhibitor

## Abstract

Peritoneal metastasis from gastrointestinal cancers has poor outcomes, and whole-abdominal radiotherapy (RT) is limited by toxicity to radiosensitive organs. Spatially fractionated radiotherapy (SFRT) delivers heterogeneous doses, potentially sparing normal tissue while stimulating antitumor immunity. This study aimed to evaluate the efficacy, safety, and immune modulation of SFRT combined with PD-L1 blockade in a syngeneic peritoneal metastasis model. Mouse-specific grid collimators (2–4 mm) were fabricated and tested. CT26-luc cells were injected intraperitoneally, followed by conventional RT (CONV) or 3 mm SFRT at 8–16 Gy, with or without anti–PD-L1. Tumor burden was assessed by bioluminescence, survival and weight monitored, and necropsy performed to evaluate toxicity. Immune profiling of ascites and blood was performed by flow cytometry, and functional outcomes by open-field testing. The 3 mm grid achieved a peak-to-valley dose ratio of ~ 11 and was used for therapy. At 8 Gy, SFRT provided tumor control comparable to CONV RT with less weight loss. At 10 Gy, CONV RT caused early mortality, whereas SFRT maintained 100% survival. SFRT plus anti–PD-L1 induced near-complete tumor regression, reduced ascites, and preserved intestinal length. Immune analysis showed increased CD4⁺/CD8⁺ T cells and M1 macrophages with SFRT; combination therapy further expanded T cells and yielded the lowest neutrophil-to-lymphocyte ratio. Functional testing demonstrated improved locomotion with SFRT plus anti–PD-L1. SFRT combined with PD-L1 blockade achieves potent tumor control, reduces toxicity, and enhances physical performance, supporting its potential as a safer immuno-radiotherapy strategy for peritoneal metastasis.

**Supplementary Information:**

The online version contains supplementary material available at https://doi.org/10.1007/s00262-026-04505-w.

## Introduction

Gastrointestinal (GI) tumors represent a diverse group of malignancies that arise from the GI tract (esophagus to anus) as well as from accessory digestive organs such as the liver and pancreas. These tumors account for more than 25% of all cancers globally, with many individual cancer types ranking among the top ten in incidence. Early diagnosis and advances in treatment–including screening, endoscopic techniques, and multimodal therapies–have markedly improved survival outcomes [1]. In particular, the integration of surgery, radiation therapy (RT), and systemic therapies has enabled some patients with distant metastases to achieve long-term survival or even potential cure [2, 3].

Peritoneal metastasis, however, remains a relatively common and devastating manifestation of GI cancers. Standard chemotherapy offers limited benefit, with median survival typically ranging from 12 to 18 months [4]. For patients without distant metastasis, aggressive approaches such as cytoreductive surgery combined with hyperthermic intraperitoneal chemotherapy (HIPEC) can provide modest survival benefit, with long-term survival achieved in only 30–40% of highly selected cases [5–8]. RT in this setting is generally limited to palliative purposes because of the risk of severe toxicity to adjacent radiation-sensitive organs, including the stomach, intestines, liver, and kidneys [9].

Immune checkpoint inhibitors (ICIs) have recently transformed the therapeutic landscape of oncology. Although objective response rates are modest–approximately 20%—responders often experience durable tumor control, supporting the expanding use of ICIs across cancer types [10]. In GI cancers, patients with high microsatellite instability (MSI-H) demonstrate superior response rates and survival with ICIs compared to chemotherapy, but this subset represents only 5–10% of the overall patient population [11]. For example, a study conducted at our institution in advanced gastric cancer demonstrated a 50% response rate among MSI-H patients (~ 10%) and Epstein-Barr virus (EBV)-positive patients (~ 7%), whereas the response rate in microsatellite-stable (MSS) patients was below 10% [12]. These findings underscore that the durable benefit of ICIs is largely confined to immunologically “hot” tumors.

Accumulating preclinical and clinical evidence suggest that combining RT with ICIs can potentiate antitumor efficacy. Our previous research demonstrated improved outcomes not only within the irradiated tumors but also in distant, non-irradiated lesions in a syngeneic liver cancer model, consistent with the so-called “abscopal effect” [13]. This phenomenon is thought to result from immunogenic cell death, whereby radiation promotes the release of tumor neoantigens and activates innate immune pathways, such as the stimulator of interferon genes (STING) signaling cascade, through the generation of damage-associated molecular patterns [14]. Furthermore, clinical studies have reported that patients with prior RT derive greater benefit from ICIs in liver and gastric cancers, even in contexts traditionally associated with poor ICI efficacy, such as sarcopenia or high neutrophil-to-lymphocyte ratio (NLR) [15–17].

Unlike conventional (CONV) RT, which delivers a uniform dose across the entire tumor volume, spatially fractionated radiotherapy (SFRT) delivers in a heterogeneous manner, creating alternating regions of high- and low-dose exposure within the target [18, 19]. This non-uniform dose distribution can be achieved using different techniques, including grid therapy, which employs a lattice-like beam arrangement, and lattice RT, which delivers high-dose “vertices” embedded within a lower-dose field. More recently, minibeam and microbeam RT approaches have been developed, in which arrays of parallel narrow beams provide spatial fractionation at submillimeter scales.

A growing body of preclinical and clinical evidence indicates that SFRT exerts distinct biological effects compared to CONV uniform RT. High-dose foci induce immunogenic cell death and vascular disruption within the tumor, while the surrounding low-dose regions help preserve immune cell infiltration and normal tissue integrity. These spatial dose variations have been shown to enhance bystander and abscopal effects, making SFRT particularly promising when combined with ICIs [20, 21]. Clinically, SFRT has demonstrated potential benefits in the treatment of bulky, radioresistant, or otherwise intractable tumors, including sarcomas [22, 23]. Because of its ability to reduce normal tissue complications while eliciting robust immune-mediated tumor control, SFRT may be especially advantageous in the setting of peritoneal metastases, where the close proximity of radiosensitive organs traditionally limits the safe use of RT.

Given these advantages and the unmet need for effective therapies in GI tumors with peritoneal metastasis, the present study was designed to evaluate the therapeutic potential of combining SFRT and ICIs in a preclinical peritoneal metastasis model.

## Materials and methods

### SFRT design

Grid collimators were custom-designed for use with an orthovoltage X-ray irradiator (X-Rad 320, Precision X-Ray, North Branford, CT, USA), operating at 320 kVp and 12.5 mA with dedicated F2 filters (1.5 mm aluminum, 0.25 mm copper, and 0.75 mm tin). To maximize the peak-to-valley dose ratio (PVDR), the collimators were fabricated from 5-mm-thick lead, providing a transmission factor of less than 10^–2^ [24]. A dedicated mount with a leg length of 1.5 cm was developed to ensure proper positioning of the collimators above the mice.

The collimator employed a perpendicular slit arrangement, in which the slit and gap widths were identical (2, 3, or 4 mm), producing a two-dimensional grid-shaped radiation field (Fig. S1a). The overlapping regions resulted in a total effective lead thickness of 10 mm. To improve experimental efficiency, the design enabled simultaneous irradiation of up to three mice.

### Radiation dosimetry

Dosimetric characterization of the grid collimators were performed using Gafchromic EBT-3 film and solid water phantoms (Fig. S1b). Irradiation time for the SFRT experiments was determined by comparing dose measurements obtained with the open field and grid collimator, ensuring equivalent delivered dose at a depth of 5 mm, corresponding to the approximate target location in mice. For all measurements, the grid collimator was placed directly on the phantom surface. Peak dose, valley dose, and PVDR were quantified from film dosimetry in all experimental conditions.

### Mice

Six-week-old male BALB/c mice were purchased from Orient Bio Inc. (Gyeonggi-do, Korea). All animal procedures and protocols were approved by the Institutional Animal Care and Use Committee (IACUC) of the Samsung Biomedical Research Institute and were performed at the Preclinical Center of Samsung Medical Center.

### Cell culture

CT26.WT-Fluc-Neo cells (CT26-luc), a mouse colorectal carcinoma cell line transduced with firefly luciferase, were purchased from Imanis Life Sciences (Rochester, MN, USA). Cells were cultured in Dulbecco’s Modified Essential Medium (DMEM; Gibco, Grand Island, NY, USA) supplemented with 10% fetal bovine serum (FBS; Gibco), 100 U/mL penicillin and 100 U/mL streptomycin (Gibco), and 0.4 mg/mL geneticin (10131-035, Gibco) at 37 °C in a humidified incubator with 5% CO_2_.

### Establishment of the peritoneal metastasis model

A peritoneal metastasis model was established by intraperitoneal injection of 5 × 10^5^ CT26-luc cells into BALB/c mice on day 0. The tumor cell suspension was injected at a single site in the lower right quadrant of the abdomen. To verify the initial tumor distribution, bioluminescence imaging was performed on day 1 post-injection (Fig. S2a), which confirmed the natural dispersion of cells throughout the peritoneal cavity. To further minimize potential confounding effects, all injections were performed by a single experienced researcher to ensure technical consistency in volume and depth. Following intraperitoneal tumor inoculation, 75 mice were randomly assigned to 25 experimental groups (n = 3 per group) based on their initial bioluminescence signal intensity to ensure an equivalent baseline tumor burden across all cohorts. The sample size was optimized to ensure simultaneous irradiation of three animals using a custom grid collimator, thereby maximizing dose homogeneity and accuracy while adhering to the Reduction principle of the 3Rs. All procedures, including group allocation, treatment, and data analysis, were conducted in a blinded manner.

Mice were subsequently administrated either an anti–PD-L1 antibody (αPD-L1) (100 μg per mouse; clone 10F.9G2, Bio X Cell, Lebanon, NH, USA) or an isotype-matched IgG control antibody (100 μg per mouse; clone LTF-2, Bio X Cell) via intraperitoneal injection on days 4 and 7. This sequence (RT followed by ICI) was selected to exploit the radiation-induced in situ vaccine effect for optimal immune priming [25]. The 100 μg dose was finalized through preliminary titration studies to ensure maximal efficacy and receptor saturation within the peritoneal cavity.

### Radiation treatment

On day 4 after tumor cell inoculation, mice were anesthetized and irradiated with 8, 10, 12, 14, or 16 Gy X-rays using an X-Rad 320 irradiator. The dose range and specific thresholds were determined based on preliminary dose-finding experiments (Fig. S2b). Specifically, 8 Gy was selected as a sub-lethal baseline dose for comparison, while 10 Gy was identified as the critical lethal threshold for conventional whole-abdominal irradiation due to acute gastrointestinal syndrome. The abdominal region was positioned within the radiation field under a grid collimator for SFRT or an open field for CONV RT.

### Ascites and whole blood sample preparation

For immune profiling, ascites and whole blood were collected on day 28. Mice were euthanized using CO_2_ asphyxiation. Ascitic fluid was obtained by carefully inserting a sterile 10 mL syringe with a 23G needle into the lower right quadrant of the abdomen and aspirating slowly to minimize tissue damage and contamination. After the initial collection, 10 mL of sterile phosphate-buffered saline (PBS) was injected into the peritoneal cavity, followed by gentle abdomen massage for 30 s. The lavage fluid was then re-aspirated to recover residual cells.

Whole blood was collected from the orbital sinus into EDTA-coated tubes to prevent coagulation. Cells from ascites and blood were subjected to red blood cell lysis using ammonium-chloride-potassium (ACK) buffer (Quality Biological, Gaithersburg, MD, USA). Samples were centrifuged at 500 × *g* for 5 min, and the supernatant was discarded. This procedure was repeated two times, if necessary, to ensure complete removal of red blood cells.

### Flow cytometry

On day 28, for immune cell distribution analysis, cells from ascites and whole blood were stained with the following antibody panels at 4 °C for 30 min: T cells with FITC anti-CD3 (145-2C11, BD Biosciences, San Jose, CA, USA), APC-Cy7 anti-CD4 (GK1.5), and V450 anti-CD8 (53-6.7); regulatory T cells (Tregs) with APC-Cy7 anti-CD4 and APC anti-CD25 (PC61), followed by fixation/permeabilization (Invitrogen, Waltham, MA, USA) at 4 °C for 20 min, and intracellular staining with PE anti-Foxp3 (MF23); neutrophils with APC-Cy7 anti-CD45 (30-F11), PerCP-Cy5.5 anti-CD11b (M1/70), and FITC anti-Ly6G (1A8); macrophages with PerCP-Cy5.5 anti-CD11b, APC anti-F4/80 (T45-2342), and PE-Cy7 anti-CD206 (MR6F3, eBioscience, San Diego, CA, USA). All antibodies were used at a 1:100 dilution.

### In vivo* imaging system (IVIS)*

Peritoneal metastases of CT26-luc cells were monitored using the IVIS Spectrum system (PerkinElmer, Waltham, MA, USA) on days 1, 4, 7, 9, 11, 17, 24, and 28 after tumor cell injection. For bioluminescence imaging, mice received intraperitoneal injections of XenoLight RediJect D-luciferin (150 mg/kg; Revvity, Waltham, MA, USA), and imaging was performed 5 min later. On day 11, various organs, including the stomach, pancreas, liver, spleen, kidneys, and intestines, were excised and analyzed ex vivo. Imaging was performed with automatic exposure, binning of 8, an F/stop of 1, and a field of view (FOV) of 22 cm for in vivo and 13.2 cm for ex vivo imaging. Data were analyzed using Living Image® software (version 4.7.2; PerkinElmer).

### Necropsy and tumor burden assessment

On day 28 post-tumor inoculation, mice were euthanized using CO_2_ asphyxiation for comprehensive ex vivo assessment. The entire gastrointestinal tract was meticulously isolated, linearized, and its total length (cm) was measured. Visibly distinct peritoneal tumor nodules adhered to the intestinal serosal surface were counted via macroscopic visual inspection, and the resulting intestinal tumor burden was quantified as nodules per centimeter (nodules/cm). Simultaneously, all macroscopic tumor masses and excised nodules from the peritoneal cavity were pooled and weighed to determine the total tumor weight (g). Furthermore, ascites fluid was collected from the peritoneal cavity by careful aspiration using a 1 mL syringe, and the volume (μL) was immediately measured to assess the extent of malignant effusion.

### Animal activity measurement

On day 28, locomotor activity was assessed using the open-field test with the Opto-Varimex-5 Auto Track system (Columbus Instruments, Columbus, OH, USA). Each mouse was placed in the center of the arena, and activity was recorded for 20 min. The arena was divided into the central and peripheral zones, and the following parameters were measured: ambulatory time (s), resting time (s), total distance traveled (cm), latency to enter the center (s), and number of center entries. Mouse tracking and data analysis were performed using the system’s automated software.

### Statistical analysis

Statistical analyses were conducted using GraphPad Prism 8.0 software (GraphPad Software, La Jolla, CA, USA). For data with small sample sizes, statistical significance was analyzed using the non-parametric Kruskal–Wallis test, followed by Dunn’s post hoc test for multiple comparisons. Survival rates were analyzed using the Kaplan–Meier method, and the significance of differences between survival curves was determined by the Log-rank test. Results are presented as the mean ± standard deviation (SD). Statistical significance was defined as *P < 0.05, **P < 0.01, ***P < 0.001.

## Results

### Establishment of grid SFRT and dosimetric evaluation

To determine the optimal spatial fractionation size for SFRT, mouse-specific grid collimators with slit sizes of 2, 3, and 4 mm were fabricated. Dose measurements and dosimetric evaluations for each grid were compared with open-field irradiation (Fig. S1). For the 3 mm grid, the irradiation time was approximately 1.5-fold longer than that of the open field to achieve an equivalent target dose. Table S1 summarizes the dosimetric characteristics of the grid collimators. Average peak doses for the 2, 3, and 4 mm grid collimators were 0.828, 0.966, and 0.941, respectively, with peak-to-valley dose ratios (PVDRs) exceeding 10 in all cases.

To evaluate therapeutic performance, a peritoneal metastasis model was generated by intraperitoneal injection of CT26-luc cells, followed by 8 Gy irradiation on day 4 using CONV open-field RT, 3 or 4 mm SFRT. This initial dose of 8 Gy was selected as a sub-lethal baseline, informed by preliminary dose-finding experiments (Fig. S2b), to compare the efficacy and safety of different grid configuration. Tumor growth was monitored by IVIS imaging for 11 days (Fig. S3a). Both CONV RT and 3 mm SFRT groups showed significantly greater body-weight loss compared with the sham group, with the largest reduction observed in the CONV RT group (Fig. S3b, c). Quantitative bioluminescence analysis confirmed tumor reduction in all irradiated groups, although tumor suppression was relatively less pronounced in the 4 mm SFRT group, likely due to a large proportion of the abdominal area receiving lower doses (Fig. S3d).

**Fig. 1:**
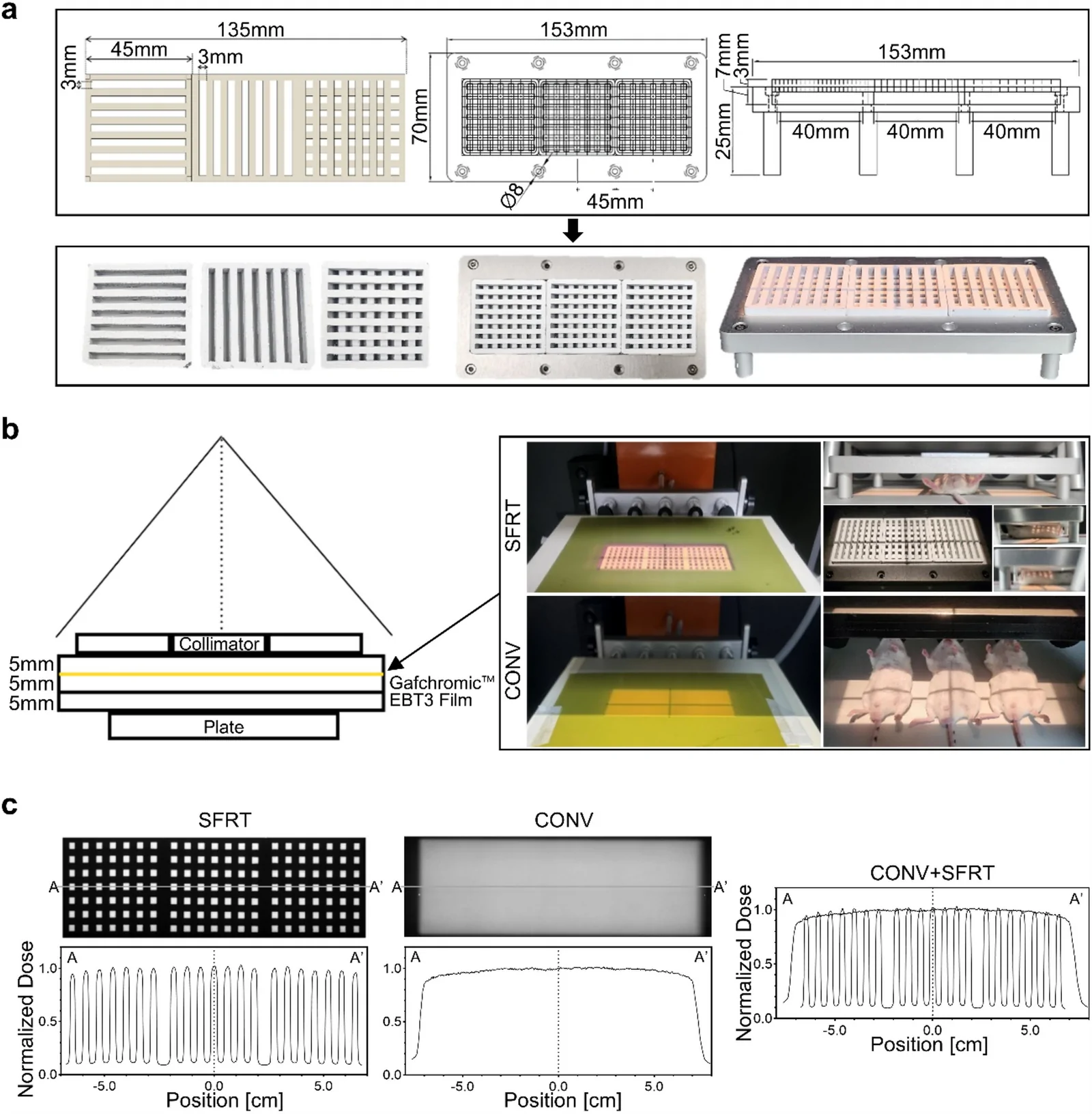
Setup of spatially fractionated radiotherapy (SFRT) and Dosimetric evaluation. **a** Design and fabrication of mouse-specific grid collimators with 3 mm slits. Representative schematics and photographs of the 3 mm collimator used for SFRT. **b** Experimental irradiation setup for SFRT and conventional (CONV) RT, showing schematic of the collimator assembly with Gafchromic EBT3 film and photographs of irradiation on mice. and SFRT irradiation setup. **c** Representative irradiated films and normalized dose profiles comparing SFRT, CONV RT, and combined SFRT/CONV distributions

Based on these dosimetric and therapeutic results, the 3 mm grid collimator was selected for subsequent SFRT experiments, as it provided favorable dosimetric properties, superior tumor-killing effect, and adequate coverage of the mouse abdomen (Fig. S4a, b). A final collimator system was fabricated from lead for radiation shielding, enabling simultaneous 3 mm grid irradiation of up to three mice (Fig. 1a). Dose profiles from the 3 mm grid collimators closely matched the open-field dose distribution, with grid peak does normalized to deliver the same target dose as CONV RT (Fig. 1b, c). Detailed dosimetry confirmed the reproducibility of the 3 mm grid collimator, with an average peak dose of 0.966, an average valley dose of 0.086, an average PVDR of 11.48 and the peak-to-peak distance of 0.61 cm (Table 1). While the PVDR ranged from 9.10 to 16.89, this variation was primarily due to localized reductions in valley doses at the peripheral edges and junctions of the custom collimator array (Fig. S4c). Importantly, because the abdominal target area was consistently positioned within the central region where dosimetric profiles remained stable and uniform, these peripheral variances did not result in differential biological outcomes or compromised reproducibility. These findings validate the suitability of the 3 mm grid design for small-animal SFRT studies.

**Table 1:** Dosimetric properties of the grid collimators with 3 mm slit and gap

	Peak dose	Valley	PVDR	Peak-to-peak distances (cm)
Median	0.977	0.090	10.98	0.61
Min	0.821	0.049	9.10	0.60
Max	1.031	0.112	16.89	0.62
Average	0.966	0.086	11.48	0.61
STD	0.049	0.015	1.80	0.00

### Therapeutic comparison between 3 mm SFRT and conventional RT

**Fig. 2:**
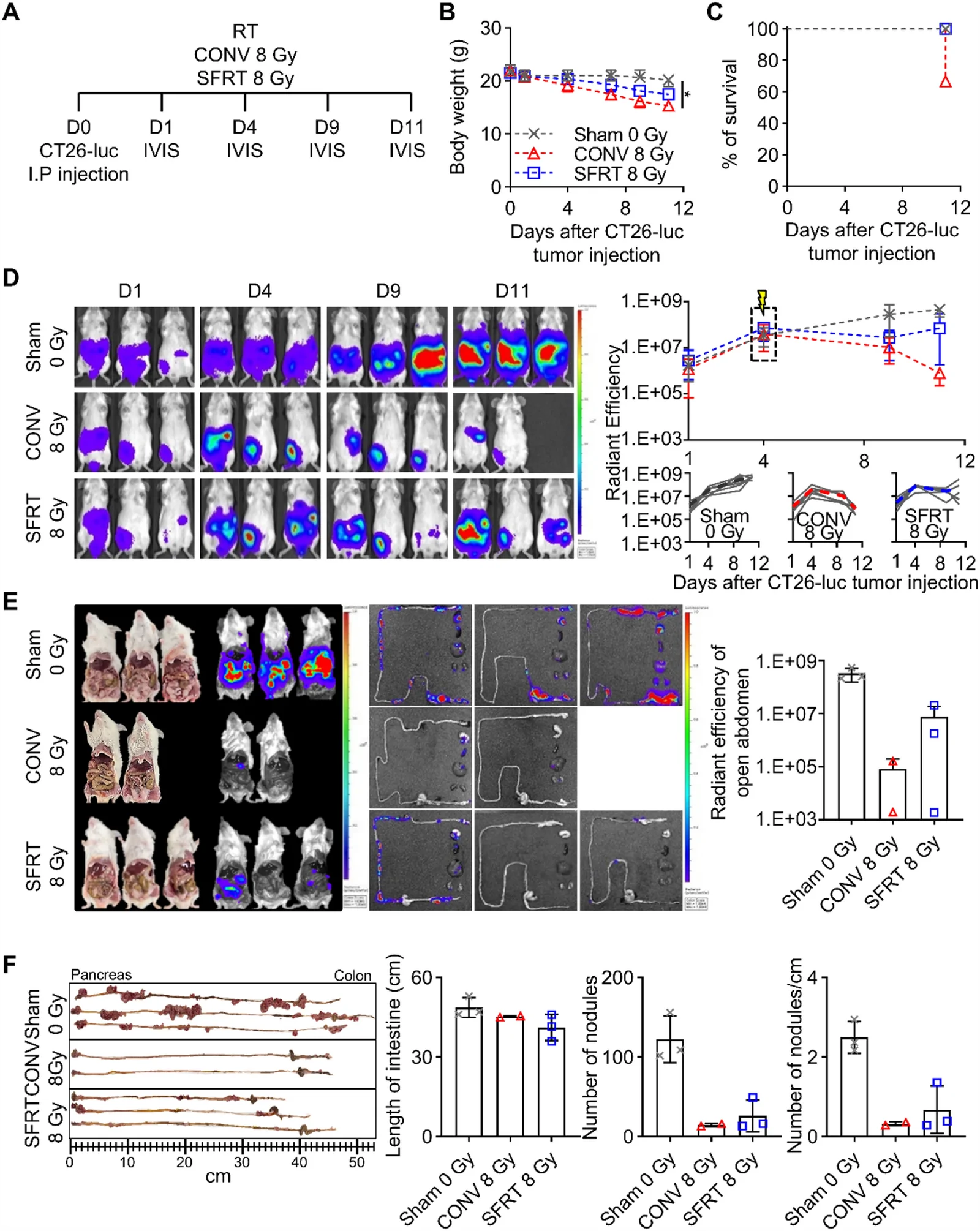
Therapeutic comparison between SFRT and CONV RT at 8 Gy in mouse peritoneal tumor model. **a** Experimental scheme showing CT26-luc cell injection and treatment schedule. **b** Body weight changes (n = 6). **c** Survival curves (n = 6). **d** Representative in vivo bioluminescence images and quantification of tumor burden (n = 6). **e** Whole-body and ex vivo bioluminescence imaging of abdominal tumors (n = 3). **f** Radiation-induced changes in gastrointestinal (GI) morphology, intestinal length, tumor nodule counts, and ascites formation (n = 3). Statistical analyses were performed using GraphPad Prism 8.0. *P < 0.05

Using the same peritoneal metastasis model, a direct comparison was performed between 3 mm SFRT and CONV RT. Mice received 8 Gy irradiation on day 4 (Fig. 2a). Over 11 days, the CONV RT group exhibited the greatest weight loss and lower survival, whereas the SFRT group showed more stable body weight and a trend toward improved survival; these observations remained consistent after pooling data from two independent experiments (n = 6; Fig. 2b, c). IVIS imaging revealed reduced abdominal tumor burden in both irradiated groups compared with controls (sham 0 Gy; non-irradiated group), although residual tumor signals remained (Fig. 2d, e). To further characterize treatment effects, necropsy was performed on day 11. Tumor dissemination to GI organs, intestinal length, and the number of tumor nodules were quantified (Fig. 2f). Both RT groups exhibited fewer visible metastases than controls, with a clear downward trend in tumor burden and nodule numbers, although the differences did not reach statistical significance. Intestinal length was slightly shorter in irradiated groups, with no statistical difference, indicating that intestinal radiation toxicity was not exacerbated in terms of gross anatomical changes. Overall, 3 mm grid SFRT achieved tumor control comparable to CONV RT while maintaining a more favorable toxicity profile at this sub-lethal dose. However, a single 8 Gy dose was insufficient for complete tumor eradication, highlighting the need for dose escalation to exploit the full therapeutic potential of SFRT or combination therapies with ICI.

### Combined effects of 8 Gy SFRT or conventional RT with ICI

**Fig. 3:**
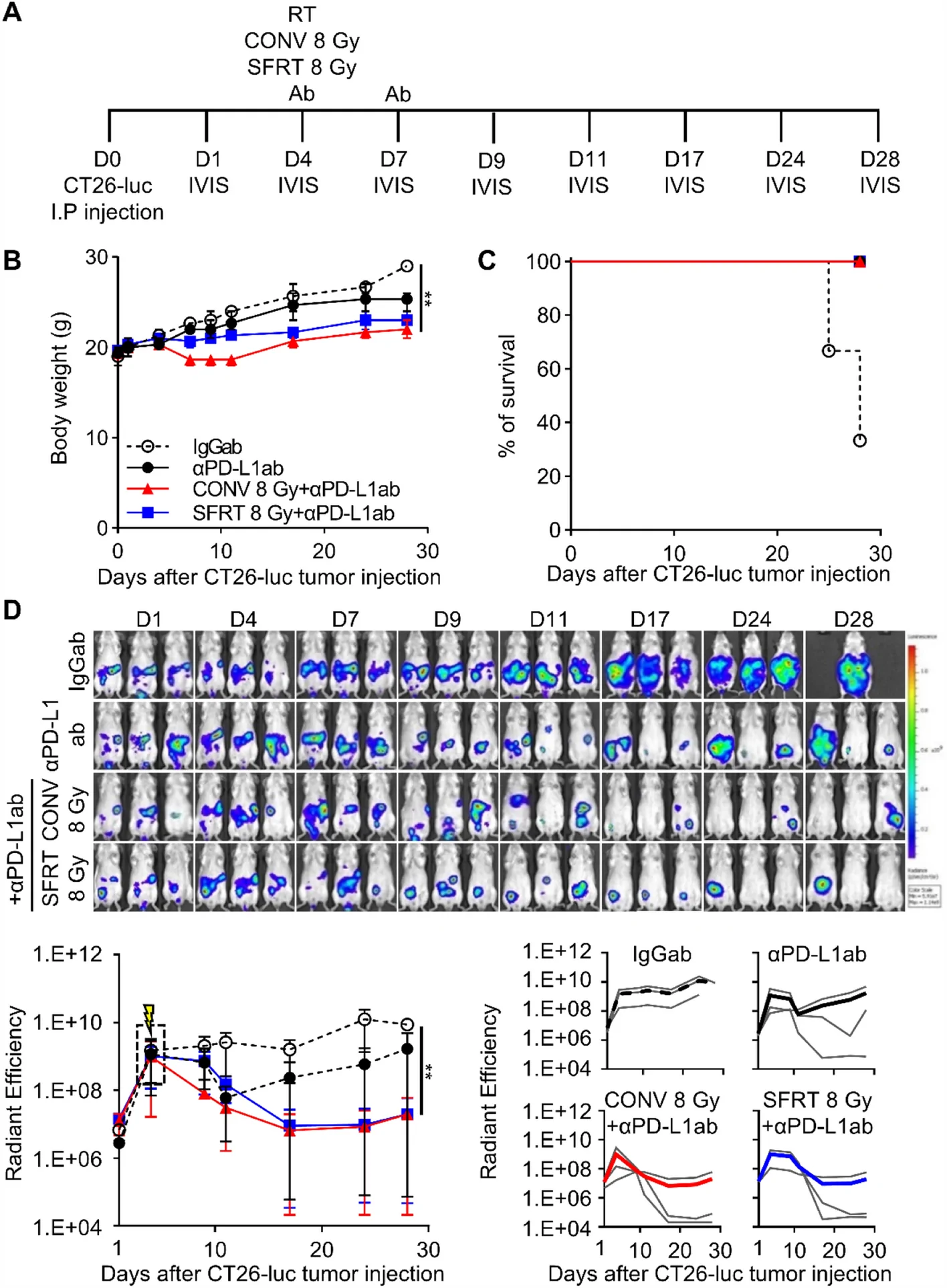
Comparative evaluation of SFRT and CONV RT at 8 Gy in combination with anti–PD-L1 antibody. **a** Experimental scheme showing CT26-luc peritoneal metastasis model, irradiation on day 4, and intraperitoneal administration of anti–PD-L1 or control IgG antibody on days 5 and 8. **b** Body weight changes (n = 3). **c** Survival curves (n = 3). **d** Representative in vivo bioluminescence images and quantification of tumor burden in each treatment group (n = 3). Statistical analyses were performed using GraphPad Prism 8.0. **P < 0.01

To evaluate the combination effects of SFRT and immune checkpoint blockade, αPD-L1 was administered after irradiation. Following establishment of the peritoneal metastasis mouse model, mice received 8 Gy of either 3 mm SFRT or CONV RT on day 4, followed by intraperitoneal injections of either αPD-L1 or control isotype IgG antibody twice at 3-day intervals (Fig. 3a). Tumor progression and systemic effects were monitored for 28 days. In controls, body weight increased over time, likely due to tumor growth and ascites accumulation, and survival decreased as tumor burden became excessive (Fig. 3b, c). By contrast, all irradiated groups maintained 100% survival by day 28. Mice treated with CONV RT + αPD-L1 showed the most pronounced transient weight loss after treatment, whereas SFRT + αPD-L1 and αPD-L1 monotherapy groups exhibited milder weight loss. IVIS imaging revealed uncontrolled tumor progression in controls, partial tumor suppression with αPD-L1 monotherapy, and strong suppression with either SFRT + αPD-L1 or CONV RT + αPD-L1, with no significant difference between the two combination groups (Fig. 3d). Importantly, the tumor burden in both RT + αPD-L1 groups remained lower than in αPD-L1 monotherapy even after regrowth. Taken together, these results indicate that αPD-L1 alone provides limited tumor control, whereas RT combination—either SFRT or CONV RT—achieves stronger, more durable tumor suppression and complete survival.

### Efficacy and toxicity of SFRT at 10 Gy or higher versus CONV RT combined with ICI

**Fig. 4:**
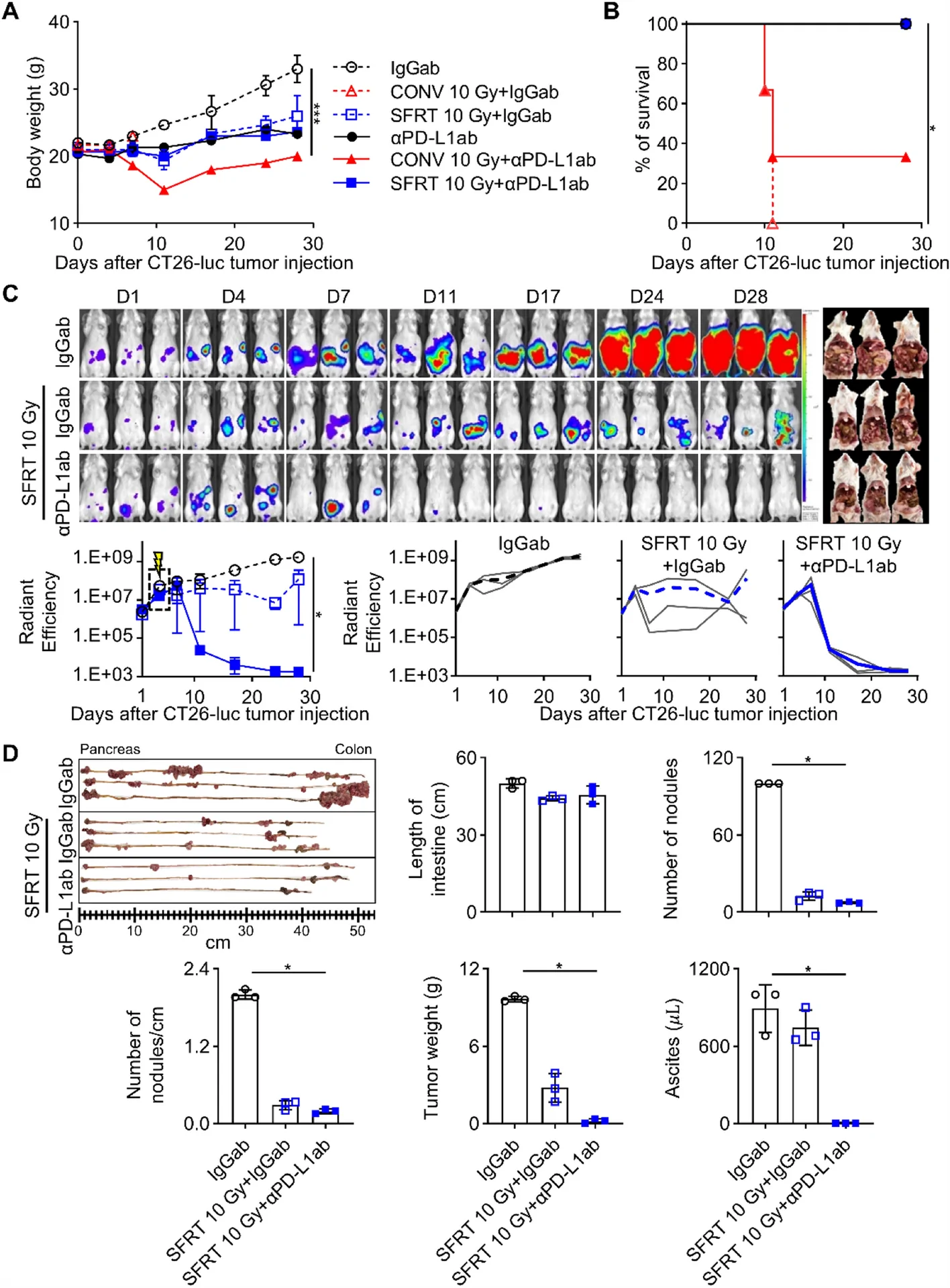
Comparative evaluation of SFRT and CONV at 10 Gy with or without anti–PD-L1 antibody. **a** Body weight changes (n = 3). **b** Survival curves (n = 3). **c** Representative in vivo bioluminescence images and quantification of tumor burden (n = 3). **d** Radiation-induced effects on abdominal organs, including GI morphology, intestinal length, tumor nodules, tumor weight, and ascites accumulation (n = 3). Statistical analyses were performed using GraphPad Prism 8.0. *P < 0.05, ***P < 0.001

To better distinguish intestinal toxicity between CONV RT and SFRT, we escalated the radiation dose to 10 Gy. All mice in the CONV RT group died within 7 days after irradiation, and only one mouse survived in the CONV RT + αPD-L1 group, which exhibited the greatest weight loss, identifying this dose as a critical lethal threshold for conventional treatment in our whole-abdominal irradiation model (Fig. 4a, b). These acute mortalities were attributed to GI syndrome, as evidenced by rapid weight loss exceeding 20%. While direct histology was not performed, these observations align with our previous reports where 10 Gy abdominal irradiation induced crypt and villus destruction within 3.5 days, leading to fatal mucosal failure [26]. In contrast, all SFRT-treated mice, either alone or combined with αPD-L1, maintained 100% survival for 28 days. Because of the poor survival observed in the CONV RT groups, they were excluded from IVIS analysis. Tumor burden continued to increase in controls, whereas SFRT alone partially suppressed growth (Fig. 4c). The combination of 10 Gy SFRT + αPD-L1 resulted in near-complete disappearance of tumor signals. Based on gross examination, intestine length was slightly reduced only in the SFRT group, whereas the number of tumor nodules and total tumor weight were markedly decreased in both SFRT groups compared with controls, with no significant difference between them (Fig. 4d). Notably, ascites volume was significantly lower in the SFRT + αPD-L1 group compared to the control IgG group, whereas the SFRT alone group did not reach statistical significance, suggesting that addition of αPD-L1 further reduced tumor-associated ascites. These results demonstrate that SFRT at 10 Gy combined with αPD-L1 provides a superior therapeutic index compared with CONV RT, achieving complete survival by bypassing the lethal dose threshold of conventional treatment.

To further confirm the safety and efficacy of combining SFRT with ICI, we evaluated SFRT at doses higher than 10 Gy. All mice in the CONV RT + αPD-L1 group died within 10 days after irradiation, whereas SFRT-treated mice at 12–16 Gy experienced transient body-weight loss but 100% survival through day 28 (Fig. S5a, b). This extended safety margin allowed for substantial dose escalation, and IVIS imaging further demonstrated that when combined with αPD-L1, higher doses of SFRT resulted in progressively greater tumor regression, indicating a dose-dependent enhancement of antitumor efficacy facilitated by superior normal tissue sparing (Fig. S5c).

### Immune profiling after 10 Gy SFRT with ICI

**Fig. 5:**
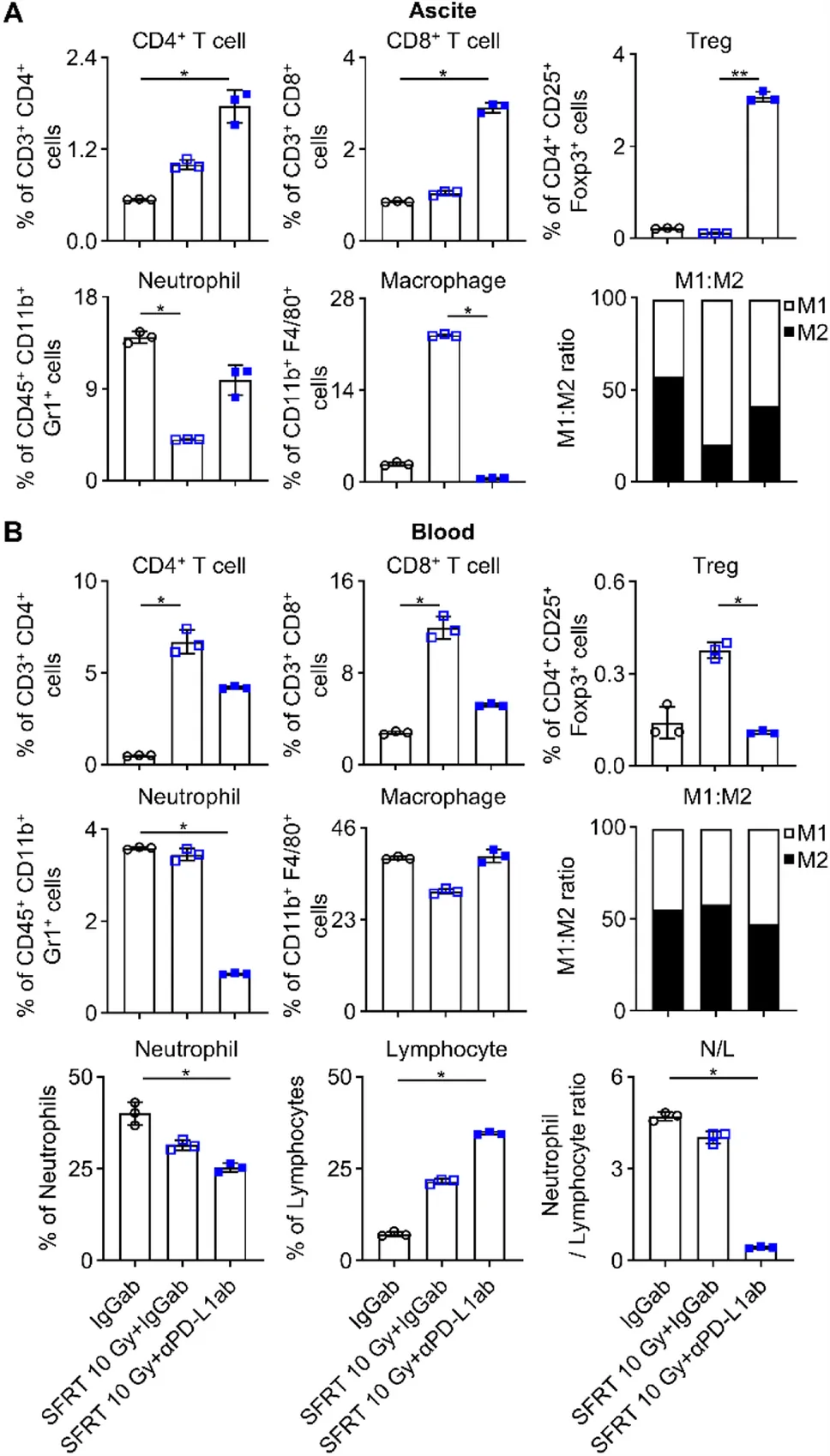
Immune profiling after 10 Gy SFRT with or without anti–PD-L1 antibody. **a** Analysis of immune cell populations in ascites. Top panels: percentage of CD3^+^CD4^+^ T cells (left), CD3^+^CD8^+^ T cells (middle), and CD4^+^CD25^+^Foxp3^+^ regulatory T cells (right) (n = 3). Bottom panels: percentage of CD45^+^Ly6G^+^CD11b^+^ neutrophils, (left), CD11b^+^F4/80^+^ macrophages (middle) and the ratio of M1(CD11b^+^F4/80^+^CD206^+^) and M2 (CD11b^+^F4/80^+^CD206^−^) (right) (n = 3). **b** Analysis of immune cell populations in peripheral blood. Top panels: percentage of CD3^+^CD4^+^ T cells (left), CD3^+^CD8^+^ T cells (middle), and CD4^+^CD25^+^Foxp3^+^ regulatory T cells (right) (n = 3). Middle panels: percentage of CD45^+^Ly6G^+^CD11b^+^ neutrophils (left), CD11b^+^F4/80^+^ macrophages (middle), and the M1/M2 macrophage ratio (right) (n = 3). Bottom panels: percentage of neutrophils (left), lymphocytes (middle), and the neutrophils-to-lymphocyte ratio (NLR) (right) (n = 3). Statistical analyses were performed using GraphPad Prism 8.0. *P < 0.05, **P < 0.01

To evaluate immune modulation induced by SFRT or SFRT combined with αPD-L1, ascites and blood were collected on day 28 for immune profiling. Ascites served as a representative surrogate for the peritoneal environment, as technical difficulties in isolating small adherent nodules and significant tumor regression in combination groups limited direct intratumoral analysis. Immune cells were isolated from ascites and blood and characterized by flow cytometry (gating strategy shown in Fig. S6). In ascites, SFRT alone did not significantly alter both CD4^+^ and CD8^+^ T cell populations compared with controls, and regulatory T cell (Treg) levels remained unchanged (Fig. 5a). However, SFRT alone significantly decreased neutrophils and increased macrophage levels, with a higher M1/M2 ratio. When αPD-L1 was added to SFRT, CD4^+^ and CD8^+^ T cells were markedly increased, accompanied by a significant rise in Tregs. In this combination group, neutrophil reduction was less pronounced, macrophage expansion attenuated, and the M1/M2 ratio returned to baseline. In peripheral blood, SRFT alone increased CD4^+^, CD8^+^ and Treg populations; however, these T-cell subsets decreased when αPD-L1 was added showing a pattern opposite to that observed in ascites (Fig. 5b). Neutrophils, which maintained a stable level after SFRT alone, were significantly reduced with SFRT + αPD-L1, whereas lymphocyte counts increased, resulting in the lowest NLR in the SFRT + αPD-L1 group.

### Improvement of physical performance after 10 Gy SFRT with ICI

**Fig. 6:**
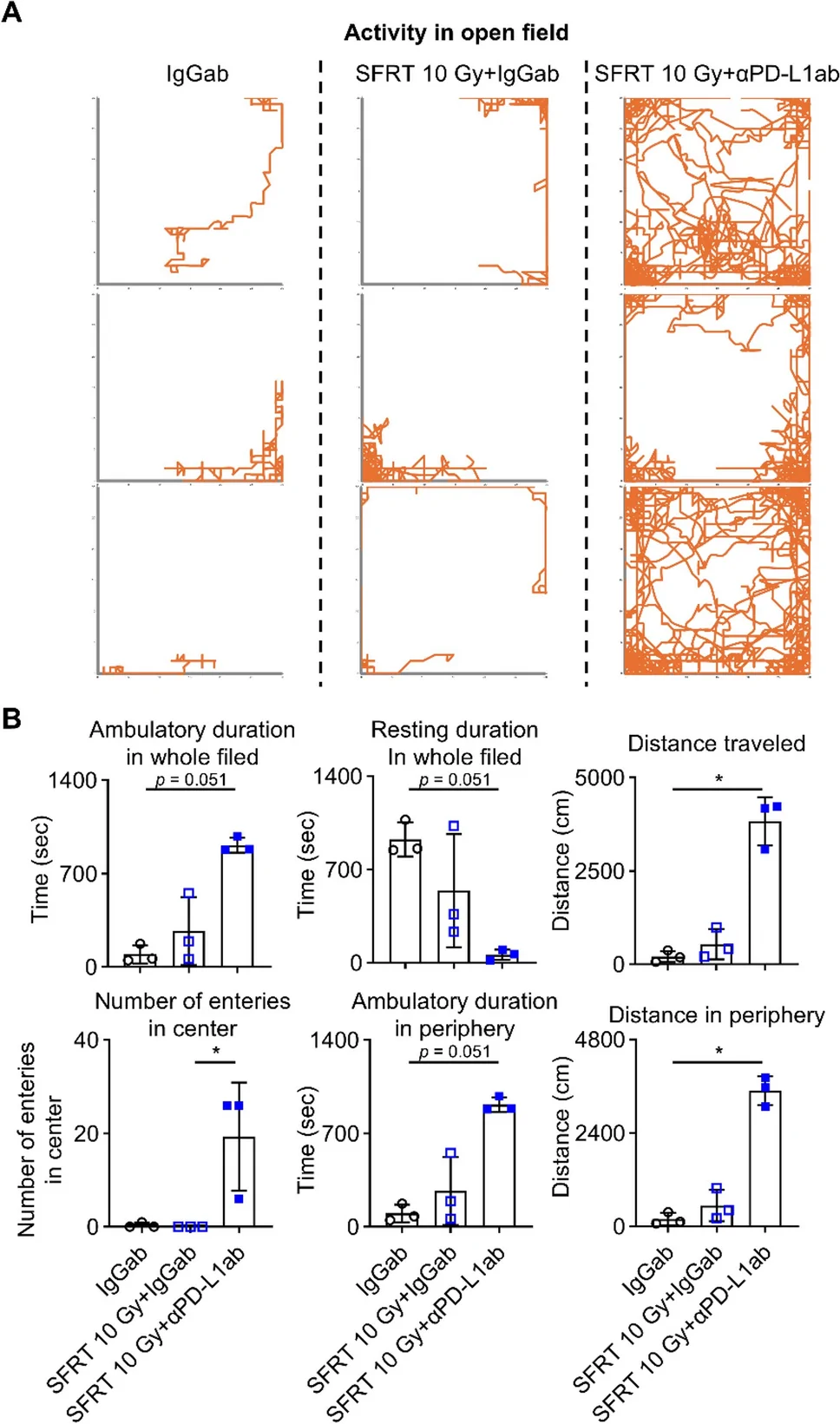
Improvement of physical performance after 10 Gy SFRT with or without anti–PD-L1 antibody. **a** Representative open-field activity maps. Red lines indicate the movement trajectories of individual mice in each treatment group (n = 3). **b** Quantification of open-field performance. Shown are ambulatory duration (whole field), resting duration (whole field), total distance traveled, number of center entries, ambulatory duration in the periphery, and distance traveled in the periphery (n = 3). Statistical analyses were performed using GraphPad Prism 8.0. *P < 0.05

An open-field test was conducted in the peritoneal metastasis mouse model to assess the impact of 10 Gy SFRT, with or without αPD-L1, on physical performance. Mice in the control and SFRT alone groups exhibited a trend toward reduced locomotion activity, with tendencies of increased resting duration and decreased ambulatory duration, distance traveled, and center entries (Fig. 6a, b). In contrast, treatment with SFRT and αPD-L1 significantly improved physical performance compared to the other groups. While the transition from CONV RT to SFRT was primarily responsible for bypassing lethal GI toxicity and ensuring survival, the addition of αPD-L1 played a decisive role in enhancing antitumor efficacy (Fig. 4). Notably, this improvement in physical activity was followed immediately by IVIS imaging and ascites analysis, suggesting that the superior locomotor function in the combination group is a direct result of the synergistic reduction in tumor burden and the complete absence of ascites (Fig. 4d). Collectively, these findings indicate that SFRT provides a safe therapeutic platform by preserving the functional integrity of intra-abdominal organs, while its combination with αPD-L1 maximizes the therapeutic index by achieving robust tumor eradication and maintaining high physical performance.

## Discussion

SFRT is an emerging radiation technique characterized by the delivery of alternating regions of high and low dose rather than relying on the traditional principle of dose fractionation, creating a spatial dose gradient within the target volume. This approach enables the delivery of high tumoricidal doses to peak regions while sparing intervening normal tissues in valley regions, thereby improving the therapeutic ratio. The PVDR is a key dosimetric parameter that reflects magnitude of dose modulation and is closely associated with both tumor control and normal tissue protection. Higher PVDR values generally correlated with greater normal tissue sparing without compromising antitumor efficacy. In our study, the 3 mm grid collimator achieved a mean PVDR of approximately 11, which is consistent with previously reported optimal ranges in preclinical SFRT studies [19, 27] and confirms the dosimetric stability and reproducibility of our setup.

It was noted that at the initial 8 Gy dose, CONV RT appeared to provide slightly better tumor control than SFRT (Fig. 2e). This may be attributed to the 1.5-fold longer irradiation time required for SFRT due to the reduced fluence through the custom grid collimator. Mechanistically, such prolonged delivery can facilitate the intra-fraction repair of sublethal damage, potentially reducing the biological effectiveness of a given dose compared to the rapid delivery of CONV RT. However, the primary advantage of SFRT lies in its superior normal tissue sparing, which offsets this minor loss in per-Gy efficacy by enabling safe dose escalation. As demonstrated in our high-dose cohorts (12–16 Gy), the escalated dose delivered via SFRT overcomes the detrimental effects of prolonged irradiation time and achieves superior overall therapeutic outcomes that are unattainable with CONV RT due to its lethal toxicity.

Given the presence of multiple radiosensitive organs in the peritoneal cavity, whole-abdominal CONV RT carries a high risk of GI and systemic toxicity [28]. To evaluate whether spatial fractionation could maintain tumor control while protecting normal tissues, we employed a syngeneic CT26 colon cancer peritoneal metastasis model, which closely recapitulates the immune-competent tumor microenvironment [29]. This model enabled us not only to compare SFRT with CONV RT for therapeutic efficacy and toxicity but also to assess immune modulation in a physiologically relevant setting. Dose-escalation experiments identified 10 Gy as a critical lethal threshold for CONV RT, reflecting the steep dose–response relationship of intestinal radiation damage (Fig. S2b). This abrupt transition from survival at 8 Gy to near-complete mortality at 10 Gy may be exacerbated by the inflammatory peritoneal environment, which likely increases mucosal radiosensitivity. Even with α‬PD-L1, the survival of only a single mouse at 10 Gy underscores the remarkably narrow therapeutic window for conventional treatment in this sensitive setting. Furthermore, SFRT was combined with αPD-L1 based on the hypothesis that its immune cell–sparing effect would enhance checkpoint inhibitor efficacy by preserving tumor-infiltrating lymphocytes and promoting a favorable immune microenvironment [20, 30]. Our results demonstrate that SFRT, particularly at higher doses (10–16 Gy), achieved robust tumor suppression with 100% survival and only transient weight loss, whereas CONV RT caused severe weight loss and early mortality despite similar tumor reduction. These findings highlight the superior therapeutic index and safety profile of SFRT for high-dose abdominal irradiation.

Tumor control was also dose-dependent, with higher doses of SFRT combined with αPD-L1 achieving near-complete tumor regression and significant reduction of ascites volume, while preserving intestinal length. Importantly, although antitumor efficacy was comparable between SFRT plus αPD-L1 and CONV RT plus αPD-L1, SFRT minimized normal tissue damage, effectively widening the therapeutic window for peritoneal metastasis treatment. These results parallel prior findings in 4T1 bilateral tumor models, where grid RT combined with immunotherapy enhanced the abscopal effect and improved systemic tumor control [31]. Our study extends these observations to a syngeneic peritoneal metastasis setting, demonstrating not only improved systemic tumor control but also direct evidence of abdominal organ protection, underscoring the translational relevance of SRFT-based immunoradiotherapy.

Preclinical studies of SFRT, including grid, lattice, minibeam, and microbeam approaches, have consistently demonstrated the ability of high-dose focal irradiation to stimulate antitumor immunity by enhancing CD8^+^ T cell infiltration and promoting proinflammatory macrophage polarization [21, 32]. Consistent with these reports, our immune profiling confirmed that SFRT alone increased CD4^+^ and CD8^+^ T cells and promoted M1 macrophage polarization within peritoneal cavity. Beyond these peak-dose effects, the unique dosimetric profile of SFRT may offer a dual advantage through its valley doses. Recent evidence indicates that while uniform high-dose irradiation to colon tissue can dampen radiation-induced antitumor immune responses [33], low-dose intestinal irradiation (~ 1 Gy) can actually synergize with PD-L1 blockade to enhance tumor control [34]. In our model, the valley regions delivered approximately 0.8–1 Gy, falling within this beneficial low-dose range. This suggests that SFRT not only delivers tumoricidal doses to the peaks but also leverages these valley doses to prime a favorable immune environment, potentially contributing to the superior immune synergy observed with αPD-L1 compared to conventional high-dose RT.

Interestingly, the decrease in peripheral T cells alongside their significant increase in ascites suggests an active recruitment of systemic immunity to the peritoneal tumor site. This trafficking of effector cells, likely facilitated by the immune-priming effect of SFRT, underscores the conversion of the immunosuppressive peritoneal cavity into an immunologically active site. The localized increase in Tregs within the ascites is interpreted as a compensatory negative feedback mechanism in response to the robust infiltration and activation of effector T cells. Crucially, the combination therapy prevented the systemic expansion of Tregs in the blood that was otherwise triggered by SFRT monotherapy. This dissociation, alongside a lower NLR observed in the blood, confirms a global shift toward anti-tumor landscape despite local counter-regulation, indicating the resolution of systemic inflammation.

Functional outcomes supported these findings. SFRT plus αPD-L1 not only improved tumor control but also enhanced physical activity, as demonstrated by increased ambulatory duration, distance traveled, and center entries in open-field testing. Critically, this locomotive recovery strongly correlated with the reduction in tumor burden and ascites volume measured on the same day, confirming that improved activity is a holistic indicator of therapeutic success rather than a mere reduction in treatment toxicity. While the SFRT platform provided the essential safety margin to bypass lethal GI toxicity, the addition of αPD-L1 was the primary driver of functional restoration through effective tumor clearance. Despite the lack of microscopic histology, the preservation of gross anatomy and high physical performance indicate that synergistic tumor suppression was achieved while maintaining the functional integrity of the GI system, ultimately improving overall performance.

The present study is subject to several limitations. First, due to the technical complexity of the custom collimator system and the ethical principle of reducing animal usage, some immunological and functional analyses were conducted with a relatively small sample size. Although the high reproducibility across independent pilot runs provides confidence in these findings, further validation with larger cohorts is warranted. Second, technical constraints in pinpointing microscopic dose-deposition sites within moving intestinal loops under SFRT limited our histological analysis. Although total intestinal length served as a macroscopic surrogate correlating with survival and weight, histological evaluation remains the gold standard for assessing mucosal integrity. Future studies utilizing spatially resolved mapping to correlate dose gradients with crypt dynamics are thus warranted to provide mechanistic insights into the sparing effects of SFRT. Third, while ascites served as a representative surrogate for the peritoneal environment, technical hurdles in isolating pure tumor-infiltrating lymphocytes from small adherent nodules prevented a direct comparison between intratumoral and systemic immune profiles. Finally, as our findings are based on a single syngeneic colorectal cancer model using male recipient mice for a female-derived cell line, they should be interpreted as hypothesis-generating. Future studies incorporating diverse and sex-matched models, such as ovarian or gastric cancer, are warranted to confirm the generalizability of the synergistic potential of SFRT and immune checkpoint blockade.

In ovarian and primary peritoneal cancers, single-agent PD-1 blockade has shown modest activity, with the KEYNOTE-100 trial reporting an objective response rate of ~ 8% and a median progression-free survival (PFS) of about 2 months [35]. In colorectal cancer, MSI-H/dMMR tumors remain responsive to ICIs; however, recent analyses suggest that patients with peritoneal metastasis experience lower response rates and shorter PFS compared with those without peritoneal involvement, while responses can still occur[36, 37]. In conclusion, the clinical literature supports the premise that radiation-immunotherapy combinations could be particularly valuable in peritoneal metastasis by enhancing antitumor immunity while managing normal-tissue risk. Our preclinical results with SFRT plus αPD-L1 align with this rationale and provide a significant proof-of-concept for the potential of SFRT in managing peritoneal malignancies. By demonstrating that SFRT provides a safe and effective platform for combination with αPD-L1, this study establishes a foundation for expanding this approach to other peritoneal disease settings. Future efforts to validate these findings across a broader range of models will be essential to pave the way for prospective clinical trials, ultimately addressing the critical unmet needs of patients with peritoneal spread where optimizing treatment response remains a challenge.‬‬‬‬‬‬‬‬‬‬‬

## Supplementary Information

Below is the link to the electronic supplementary material.

Supplementary file1 (PDF 930 kb)

Supplementary file2 (DOCX 18 kb)

## Data Availability

All data generated and analyzed during this study are included in this published article and its supplementary files.
