# Supplementary material for: Spatially fractionated radiotherapy with PD-L1 blockade achieves superior antitumor efficacy and safety in a preclinical model of peritoneal metastasis
Source: Cancer Immunol Immunother. 2026 Aug 6;75(11):267. doi: 10.1007/s00262-026-04505-w (PMC13731542; doi:10.1007/s00262-026-04505-w)
Supplement: Supplementary file 1 [file 262_2026_4505_MOESM1_ESM.pdf]

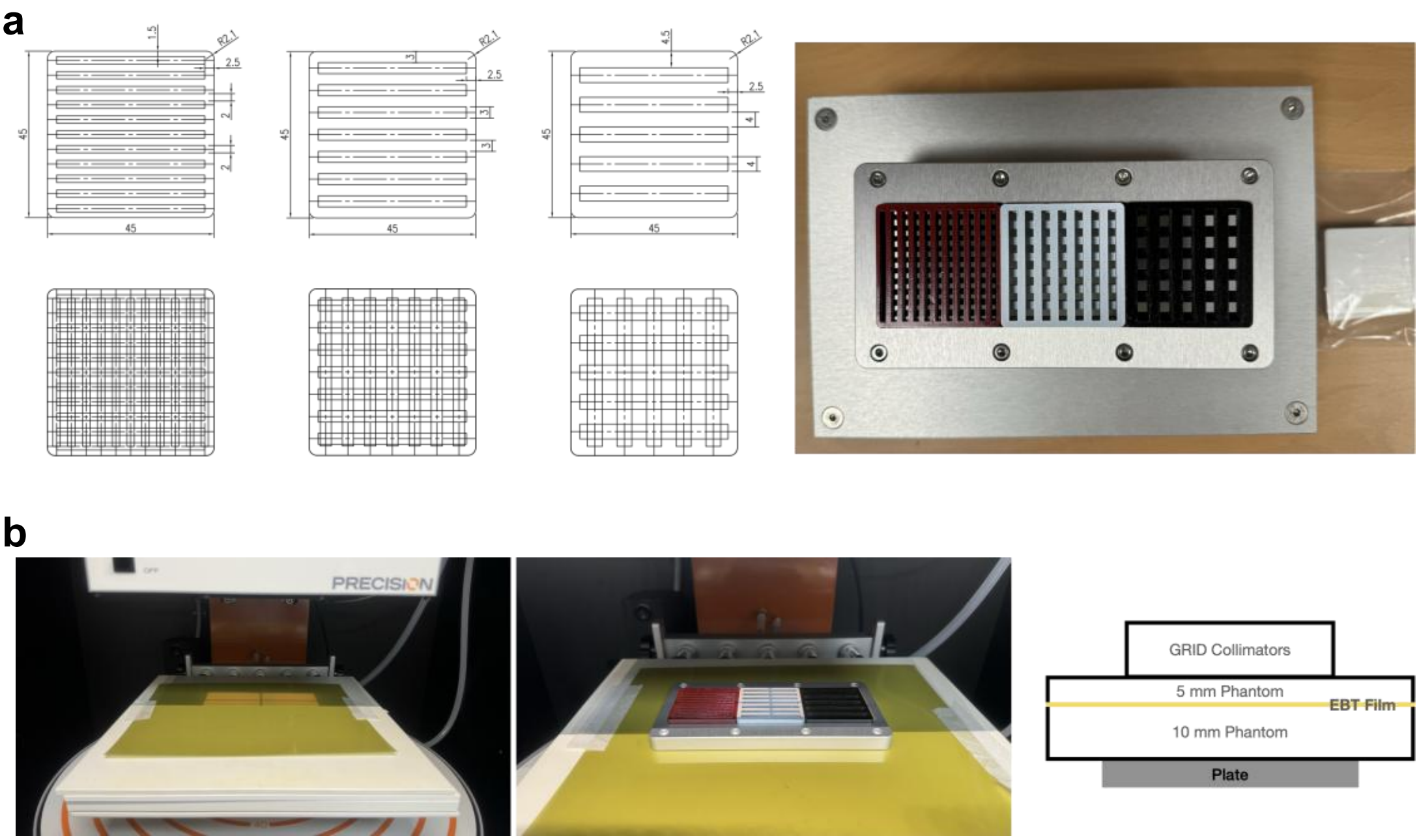

Figure S1. Collimator assembly and experimental configuration for SFRT dosimetry. (a) Mechanical drawings and an actual photograph of the custom-fabricated grid collimators with varying aperture size (2-4mm). (b) Experimental setup for dose delivery: (Left): open field for CONV RT; (Center) grid collimator for SFRT; (Right) schematic showing the vertical stack consisting of the collimator, a 5 mm buildup phantom, EBT film for dose recording, and a 10 mm backscatter phantom.

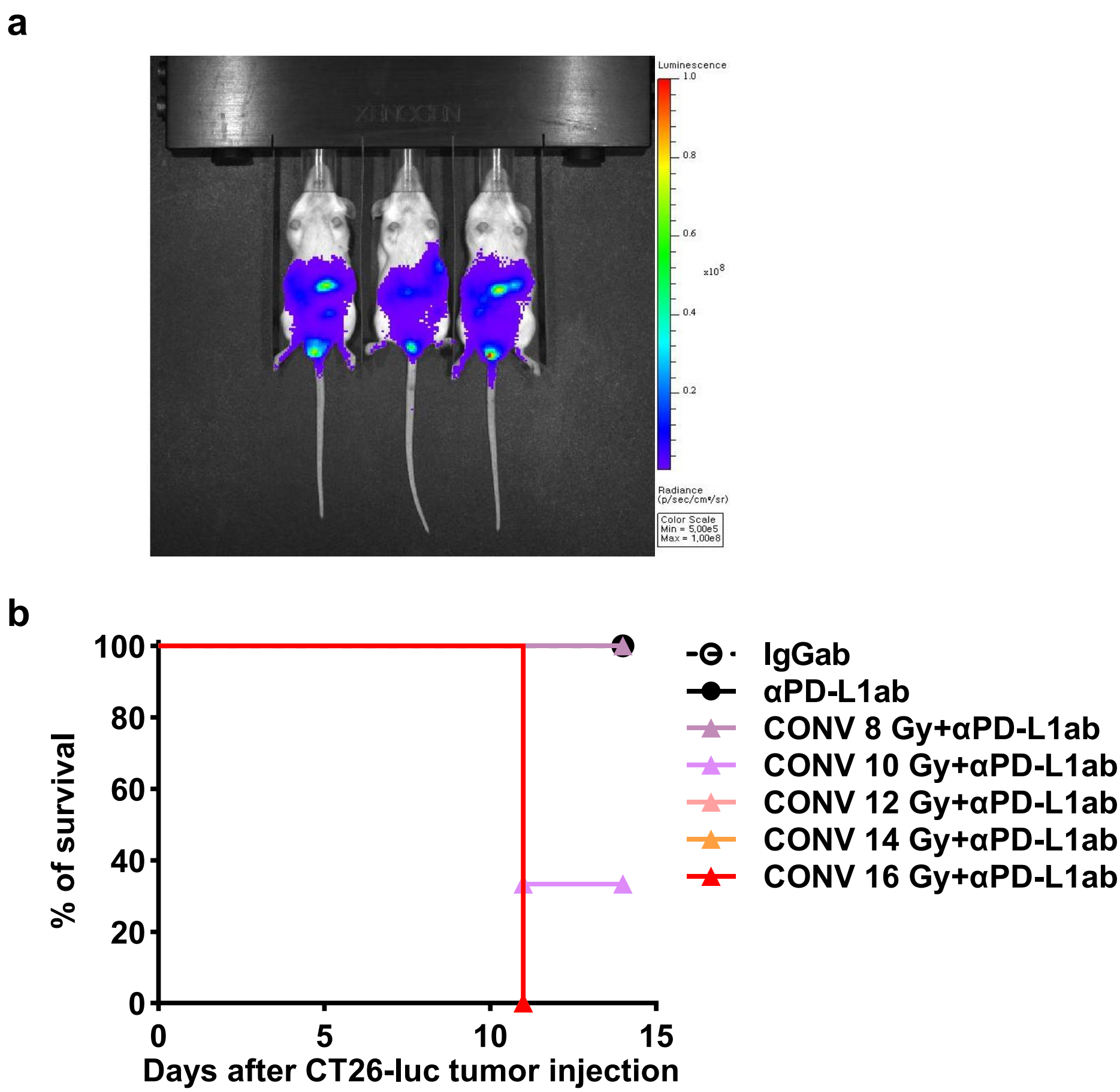

Figure S2. Initial tumor distribution and dose-dependent survival outcomes after whole-abdominal irradiation. (a) Representative bioluminescence image captured one day after the intraperitoneal injection of CT26-luc cells into the lower right abdomen. The widespread and relatively uniform bioluminescence signal across the entire abdominal region confirms the successful establishment of the peritoneal metastasis model and consistent initial tumor cell distribution prior to treatment. (b) Comparison of survival rates in CT26-luc tumor-bearing mice across different CONV radiation doses (8, 10, 12, 14, and 16 Gy) combined with anti-PD-L1. Total survival (100%) was observed in the 8 Gy group, whereas 100% mortality was reached at doses ≥ 12 Gy. Control groups (IgG and anti-PD-L1 monotherapy) are also shown.

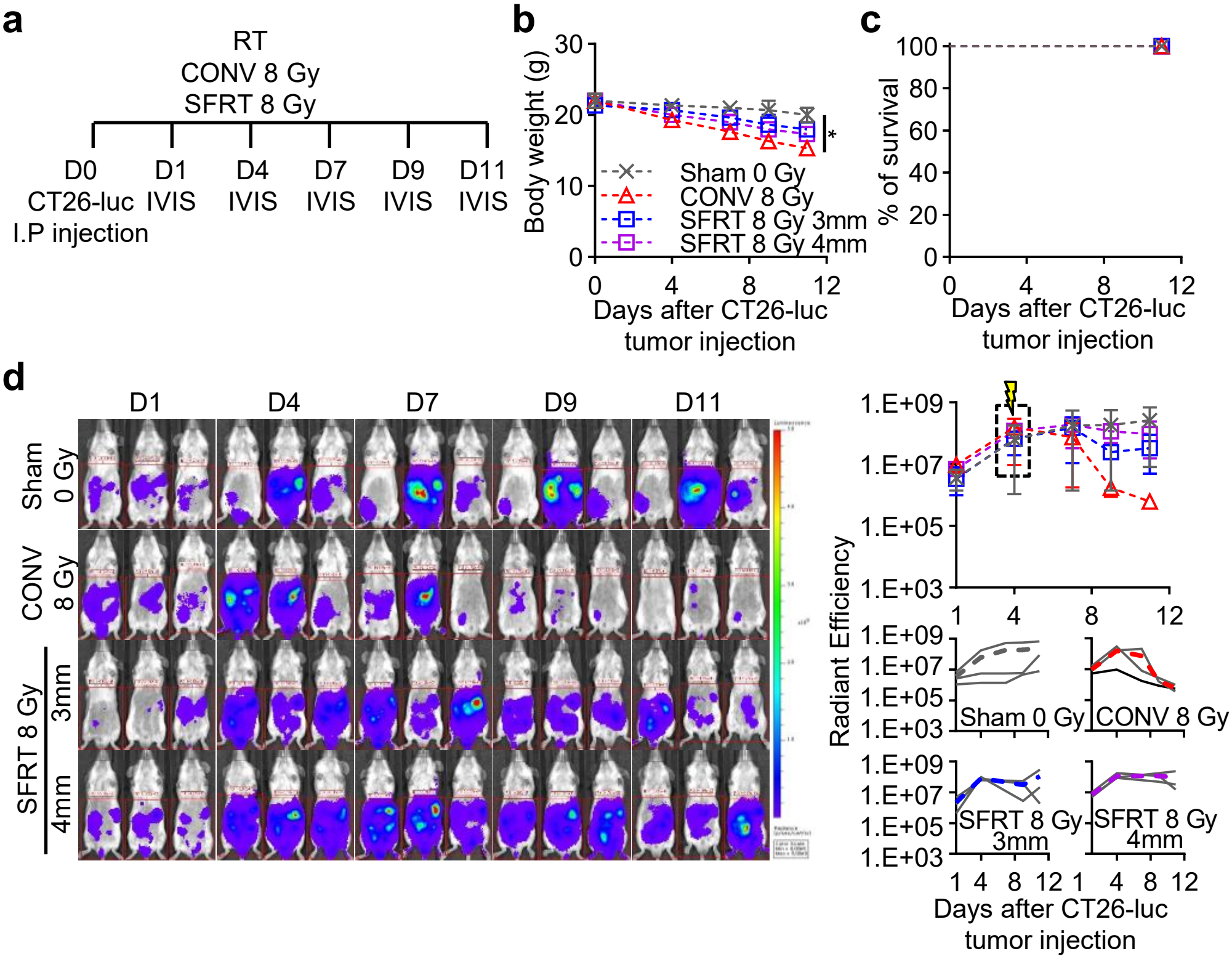

Figure S3. Comparative therapeutic efficacy and safety between 3 mm and 4 mm SFRT. (a) Experimental timeline. (b) Changes in body weight. (c) Kaplan-Meier survival curves showing 100% survival in all 8 Gy-irradiated groups. (d) Representative in vivo bioluminescence imaging (left) and quantification of tumor-derived radiant efficiency (right) over 11 days. Statistical analysis was performed using GraphPad Prism 8.0. *$P<0.05$.

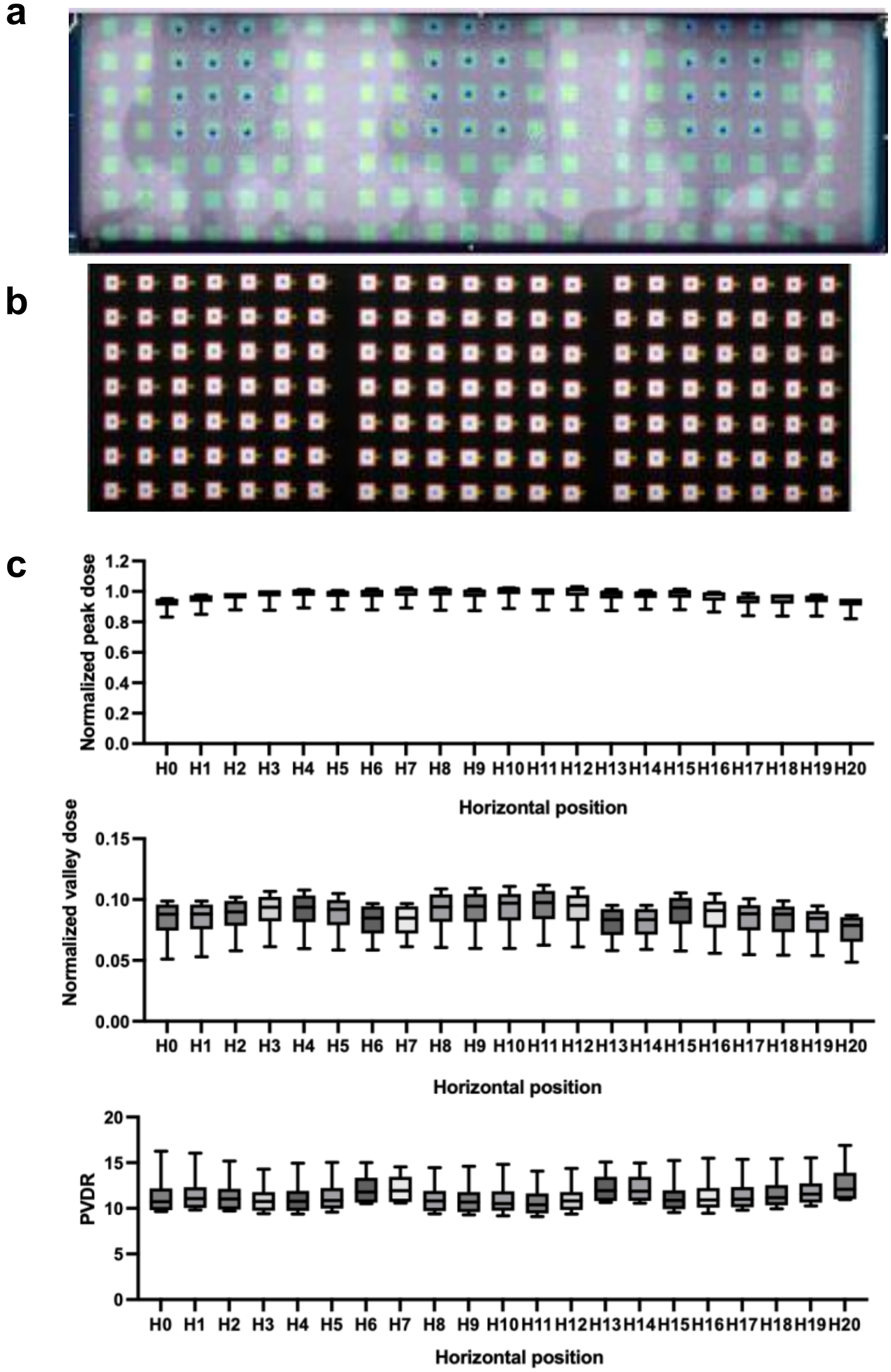

Figure S4. Identification of grid patterns and dosimetric validation. (a) Representative image of the mouse abdominal area overlaid with the identified grid pattern. Green squares and blue asterisks indicate the individual grid areas and targeted irradiation spots, respectively. (b) Automated detection of grid outlines (read) and centroids (blue) for precise targeting. (c) Dosimetric analysis across the horizontal grid positions (H0-H20). Plots show that normalized peak dose (top), normalized valley dose (middle), and peak-to-valley dose ratio (PVDR, bottom). While peak doses are uniform, PVDR increases ate the collimator edges due to reduced valley doses; however, these regions are excluded from the actual abdominal irradiation area.

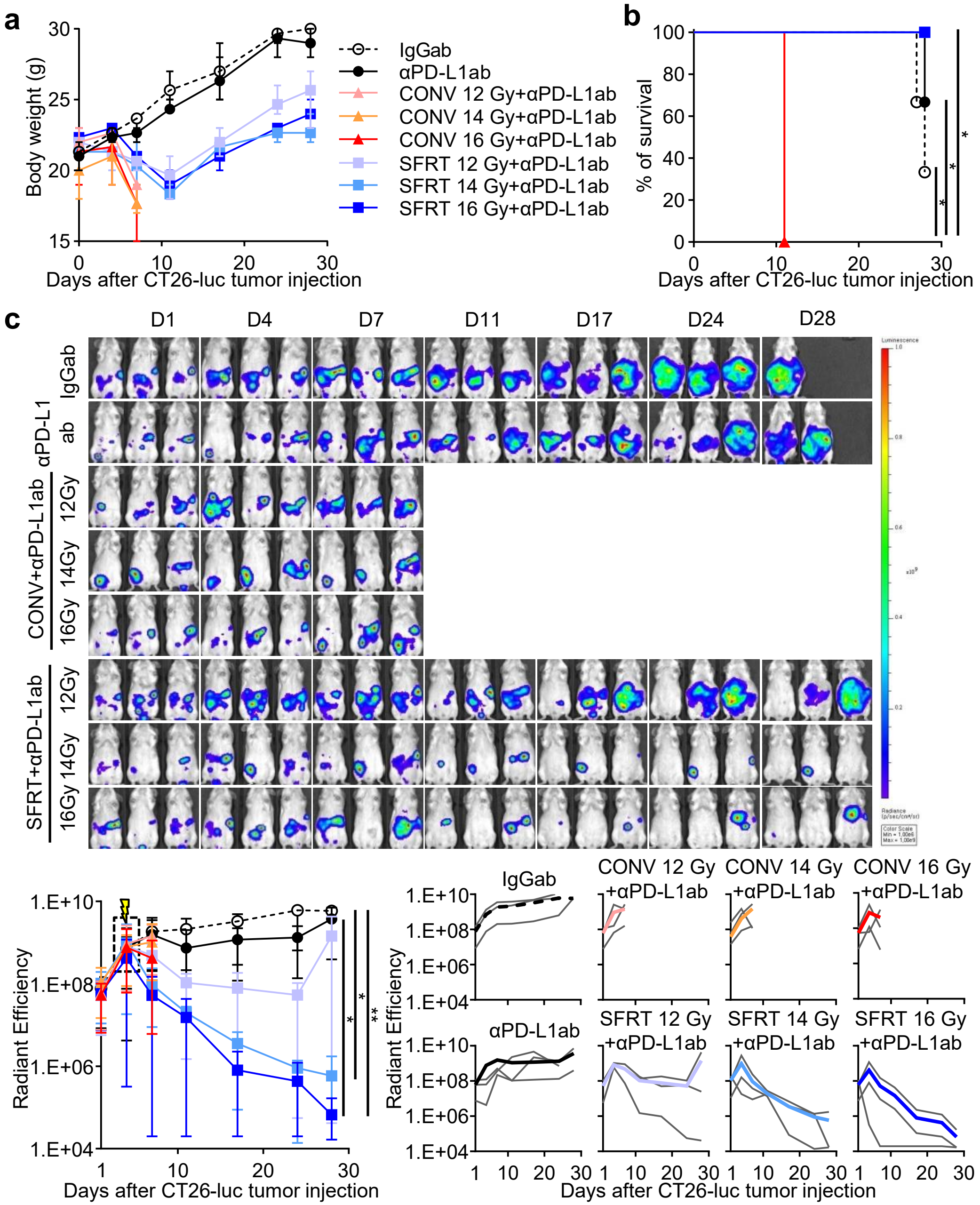

Figure S5. Comparative efficacy and safety of high-dose SFRT vs CONV RT combined with anti-PD-L1. (a) Body weight change showing acute lethality in CONV group versus recovery in SFRT groups. (b) Survival curves. CONV RT results in 100% mortality by Day 11, while SFRT significantly extended survival (*P<0.05, **P<0.01) (c) *In vivo* bioluminescence imaging and radiant efficiency quantification, demonstrating superior tumor regression in SFRT + anti-PD-L1 groups. Statistical analysis was performed using GraphPad Prism 8.0. **P* < 0.05, ***P* < 0.01.

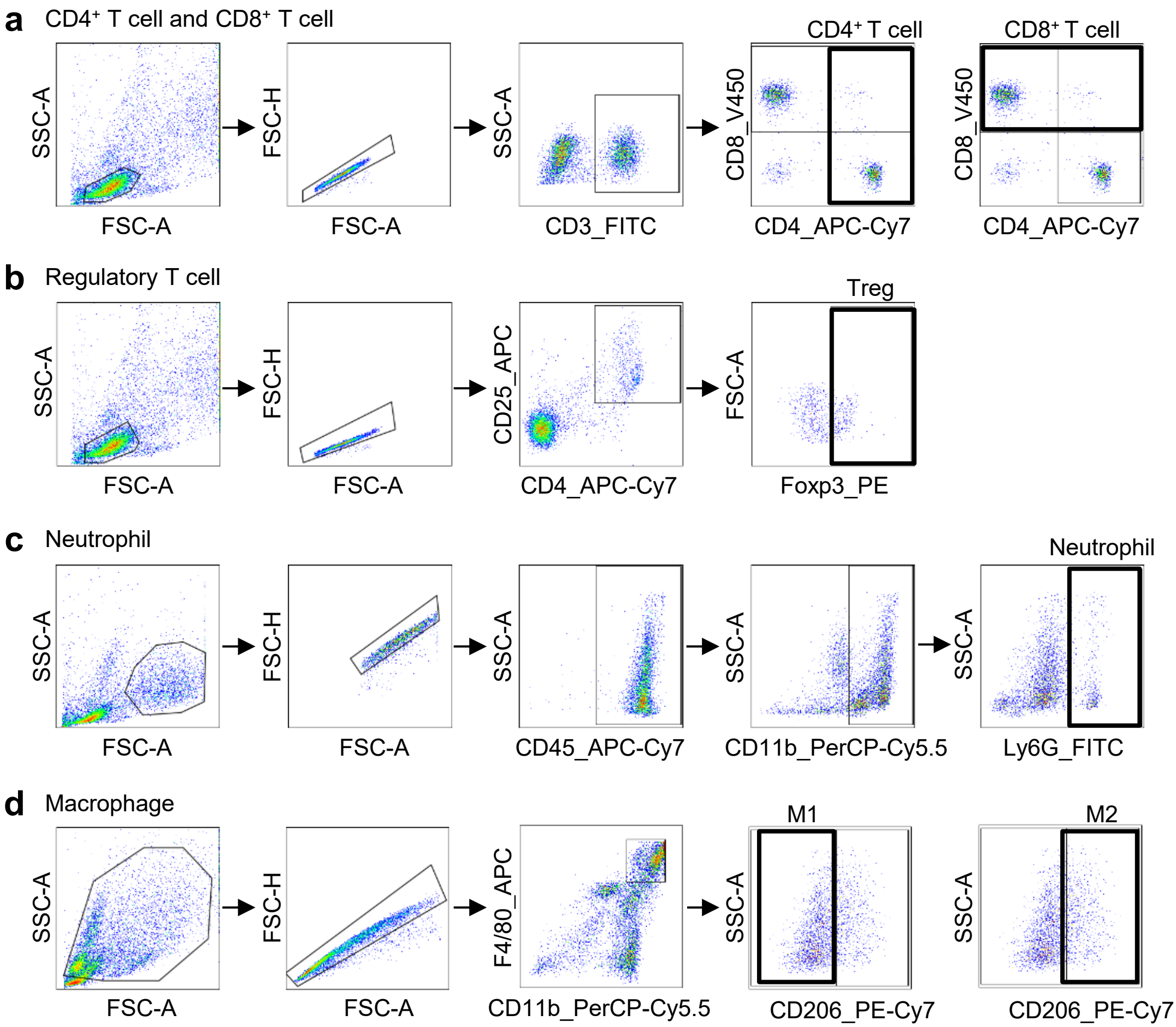

Figure S6. Flow cytometry gating strategy for immune cell profiling. Representative gating plots for identifying immune cell populations in ascites and blood. Initial gates were set on FSC/SSC to exclude debris, followed by doublet removal using FSC-H/FSC-A. (a) T cells: $CD3^+$ $CD4^+$ and $CD3^+$ $CD8^+$ T cell. (b) Regulatory T cells: $CD4^+$ $CD25^+$ $Foxp3^+$ cell. (c) Neutrophils: $CD45^+$ $CD11b^+$ $Ly6G^+$ cells. (d) Macrophages: $CD11b^+$ $F4/80^+$ cells further subdivided into M1 ($CD206^-$) and M2 ($CD206^+$) phenotypes.
