# Supplementary material for: Spatially fractionated radiotherapy with PD-L1 blockade achieves superior antitumor efficacy and safety in a preclinical model of peritoneal metastasis
Source: Cancer Immunol Immunother. 2026 Aug 6;75(11):267. doi: 10.1007/s00262-026-04505-w (PMC13731542; doi:10.1007/s00262-026-04505-w)
Supplement: Supplementary file 2 [file 262_2026_4505_MOESM2_ESM.docx]

**Table S1**. Dosimetric Characteristics of Grid Collimators (2 mm, 3 mm, and 4 mm Slit and Gap)

|  |  | Peak | Valley | PVDR | Peak-to-peak distances  (cm) |
| --- | --- | --- | --- | --- | --- |
| 2 mm | Median | 0.836 | 0.057 | 14.2 | 0.41 |
|  | Min | 0.507 | 0.024 | 11.0 | 0.37 |
|  | Max | 0.921 | 0.074 | 26.3 | 0.42 |
|  | Average | 0.828 | 0.054 | 15.2 | 0.41 |
|  | STD | 0.066 | 0.013 | 3.5 | 0.01 |
| 3 mm | Median | 0.980 | 0.062 | 15.2 | 0.61 |
|  | Min | 0.879 | 0.037 | 11.9 | 0.58 |
|  | Max | 1.021 | 0.082 | 22.9 | 0.62 |
|  | Average | 0.966 | 0.061 | 16.0 | 0.61 |
|  | STD | 0.052 | 0.014 | 3.1 | 0.01 |
| 4 mm | Median | 1.017 | 0.042 | 22.1 | 0.81 |
|  | Min | 0.649 | 0.023 | 14.7 | 0.76 |
|  | Max | 1.080 | 0.070 | 31.2 | 0.82 |
|  | Average | 0.941 | 0.044 | 21.4 | 0.79 |
|  | STD | 0.133 | 0.015 | 4.5 | 0.02 |
